# Readiness to Provide Neonatal Care Services in 208 Ethiopian Hospitals Prior to Implementation of the Saving Little Lives Program

**DOI:** 10.3390/children13040481

**Published:** 2026-03-30

**Authors:** Lamesgin Alamineh Endalamaw, Abiy Seifu Estifanos, Araya Abrha Medhanyie, Mekdes Shifeta Argaw, Abebe Gebremaraim Gobezayehu, Abebech Demissie Aredo, Znabu Hadush Kahsay, Hege Langli Ersdal, John Nutting Cranmer, Damen Hailemariam, Siren Irene Rettedal

**Affiliations:** 1 The Emory Ethiopia Office, Emory University, Addis Ababa P.O. Box 9086, Ethiopia; abebe.g.gobezayehu@emory.edu; 2 Faculty of Health Sciences, University of Stavanger, 4068 Stavanger, Norway; hege.ersdal@safer.net (H.L.E.); siren.irene.rettedal@sus.no (S.I.R.); 3 Center for Implementation Sciences (CIS) in Health, Addis Ababa University, Addis Ababa P.O. Box 1176, Ethiopia; abiy.seifu@aau.edu.et (A.S.E.); abebech.demissie@aau.edu.et (A.D.A.);; 4 Department of Reproductive, Family and Population Health, School of Public Health, Addis Ababa University, Addis Ababa P.O. Box 1176, Ethiopia; 5 MARCH Research Center, College of Health Sciences, Mekelle University, Mekelle P.O. Box 1871, Ethiopiaznabu.hadish@mu.edu.et (Z.H.K.); 6 Faculty of Public Health, College of Health Sciences, Mekelle University, Mekelle P.O. Box 1871, Ethiopia; 7 Department of Pediatrics and Child Health, School of Medicine, College of Medicine and Health Sciences, Hawassa University, Hawassa P.O. Box 1560, Ethiopia; mekdess@hu.edu.et; 8 Department of Simulation-Based Learning, Stavanger University Hospital, 4068 Stavanger, Norway; 9 Implementation Science in Global Reproductive Health (Ethiopia), Emory University, Atlanta, GA 30322, USA; john.cranmer@emory.edu; 10 Department of Health Systems and Health Policy, School of Public Health, Addis Ababa University, Addis Ababa P.O. Box 1176, Ethiopia

**Keywords:** saving little lives program, readiness, newborn care services

## Abstract

**Highlights:**

**What are the main findings?**

- Hospitals’ readiness to provide care for small and sick newborns across the continuum of care was suboptimal, with readiness observed in 59% of labor and delivery wards and 57% of neonatal care units.
- Readiness varied by hospital level, with differences noted across referral, general, and primary hospitals.

**What are the implications of the main findings?**

- There were substantial disparities across hospital levels that impact readiness to provide quality care for small and sick newborns.
- These findings indicate the need for policymakers and health system leaders to prioritize the hospital readiness for small and sick newborns across the continuum of care as a key policy and programmatic focus.

**Abstract:**

Introduction: Despite improved health service accessibility, neonatal mortality in Ethiopia remains high at 33 per 1000 live births. Thus, improving health facilities’ readiness across infrastructure, basic amenities, equipment, medications, laboratory services, Kangaroo Mother Care, infection prevention and control, staffing, and guidelines availability is critical for improving the quality of neonatal care and survival. Objective: The aim of this study was to evaluate the readiness of Ethiopian hospitals to provide services to small and sick newborns. Methods: This was a cross-sectional study including 208 hospitals across four regions in Ethiopia in 2021–2024, prior to the implementation of the Saving Little Lives program. Data was collected using an adapted World Health Organization’s Service Availability and Readiness Assessment tool and are presented using composite scores. Results: The mean composite readiness score for the 208 hospitals for providing services to small and sick newborns in labour and delivery wards was 59%, with domain-specific scores of 47% for basic amenities, 56% for essential neonatal care, and 74% for newborn resuscitation. Significant variation was seen across hospital levels, and basic amenities were available in 68%, 49%, and 43%, essential neonatal care in 68%, 81%, and 71%, and newborn resuscitation in 68%, 66%, and 50% of referral, general, and primary hospitals, respectively. The mean composite readiness score to provide newborn care in the neonatal care units was 57%. Scores varied by hospital levels, with scores of 73%, 64%, and 50% for referral, general, and primary hospitals, respectively. Domain-specific scores were 63% for basic amenities, 65% for equipment, 67% for medications, 63% for laboratory services, 25% for Kangaroo Mother Care, 68% for infection prevention and control, 55% for staffing, and 51% for guidelines availability. Functional bCPAP machines were available in 14% of labour and delivery wards and in 35% of neonatal care units. Conclusions: There was a substantial gap in readiness to provide care for small and sick newborns, and significant variations across hospital levels. Immediate actions must be taken to address the observed gaps to reach the sustainable development goal of reducing neonatal mortality to at least 12 per 1000 live births by 2030.

## 1. Introduction

Neonatal mortality remains a critical global health challenge. In 2022, 4.9 million children under five years of age died, and 47% of these deaths occurred in the first 28 days of life. Sub-Saharan Africa accounts for 57% of under-five deaths and 46% of neonatal deaths. While overall under-five mortality has declined since 1990, the proportion of neonatal deaths increased from 41% in 2000 to 47% in 2022 [1]. According to the World Health Organization (WHO), an estimated 20 million infants are born with low birth weight (LBW) each year, and 15 million are born preterm [2]. In 2020, an estimated 80% of neonatal deaths occurred among LBW infants, and nearly two-thirds of deaths were among those born prematurely [3].

Ethiopia has a high neonatal mortality rate of 33 per 1000 live births, with approximately 107,000 neonatal deaths annually, accounting for 56% of under-five deaths [4]. Complications from prematurity are the leading cause, including respiratory distress syndrome (RDS) (45%), infections (30%), and birth asphyxia (14%) [5].

Although cause-specific neonatal mortality varies by geographical area, it is largely attributed to poor quality of care at birth and during the early neonatal period, estimated to contribute to 61% of neonatal deaths [6]. With high-quality health systems, it is estimated that one million newborn deaths can be prevented each year [7]. Therefore, improving both access to and quality of care is critical and requires actions to ensure neonatal survival. The readiness of hospitals is an essential prerequisite for ensuring service quality, including infrastructure, basic amenities, equipment, medications, laboratory services, trained healthcare providers (HCPs), and guideline availability [8].

To address the stagnant neonatal mortality rate, Ethiopia has implemented key interventions such as establishing dedicated newborn wards, training HCPs, and establishing and equipping neonatal care units (NCUs) across different hospital levels. Referral hospitals are expected to offer specialized level 3 care, general hospitals to deliver level 2 care, and primary hospitals to provide basic level 1 care. All hospitals are required to have a Kangaroo Mother Care (KMC) ward for eligible preterm and LBW infants [9].

Despite improved service accessibility, persistent challenges such as uneven resource distribution, poor quality of care, low KMC coverage, low community care-seeking behavior, and shortages of essential commodities and equipment at service delivery points remain key challenges contributing to the high neonatal mortality rate in Ethiopia. Addressing these problems requires an understanding of national newborn care priorities, and comprehensive facility assessments need to be made to determine how to improve facility readiness, HCPs competencies, provider–patient interactions, and working environments [10].

From January 2021 to June 2024, the Ethiopian government with the support from the World Bank’s Global Financing Facility (GFF) implemented the Saving Little Lives (SLL) program, aiming to reduce neonatal deaths by 35% through achieving 80% coverage of evidence-based interventions targeting hospitalized preterm and LBW infants [11]. The program’s success depended on the readiness and availability of functional health infrastructures, medical equipment, and medications as well as on the availability of adequate and trained HCPs in the hospitals to deliver quality neonatal care.

This study was conducted to assess the readiness of hospitals included in the program to provide care to small and sick newborns prior to the implementation of the SLL program and generate actionable information about the readiness and availability of neonatal care services in Ethiopia.

## 2. Materials and Methods

### 2.1. Study Design, Setting, and Inclusion Criteria

This facility-based cross-sectional study was part of the SLL program that aimed at reducing the neonatal mortality rate by 35% through achieving 80% coverage of evidence-based interventions [11].

The SLL program targeted 290 hospitals, representing 82% of all hospitals in the country at the time of the study. The hospitals were spread across four regions (Oromia, Amhara, Tigray, and Southern Nations, Nationalities and People (SNNP)), covering a total population of an estimated 76.2 million and 1.2 million births (34% of national births). These hospitals were selected for SLL program implementation based on their high delivery volume, neonatal mortality rates, and in alignment with government priorities.

Among the 290 hospitals, data were not collected from 82 hospitals due to security challenges at the time of the assessment, their remote locations, or resource constraints. Therefore, data from 208 hospitals of all levels across the four regions were included in the final analysis, including 22 referral, 56 general, and 130 primary hospitals.

The SLL program was designed to be implemented in three phases, each lasting a year. Data on the readiness of hospitals to provide care for small and sick newborns were collected in phases prior to implementation of the stepped-wedge SLL program from March to May 2021, July to August 2022, and December 2023 to January 2024, including 72, 115, and 21 hospitals, respectively.

### 2.2. SLL Program Interventions

The SLL program was designed to implement evidence-based minimum care packages interventions to small and sick newborns, targeting the primary drivers of neonatal mortality: prematurity, birth asphyxia, and sepsis. Across the continuum of neonatal care, the SLL minimum care packages focused on care at birth in labour and delivery (L&D) wards, in the NCUs, and in the KMC wards.

The minimum care package at birth included birth preparation and essential newborn care, including resuscitation for asphyxiated infants, respiratory support for preterm infants, infection prevention and control (IPC), thermal care, early breastfeeding initiation, and timely safe referral. The NCU care package included IPC and sepsis management, the use of bubble CPAP (bCPAP) for infants with symptoms of RDS or gestational age < 32 weeks, thermal care, feeding support, and management of perinatal asphyxia. The KMC package consisted of exclusive breastfeeding and skin-to-skin contact for at least 8 h per day.

The SLL minimum care packages were cascaded through training and clinical mentorship to enhance HCPs’ knowledge and skills. Additionally, selected hospitals received essential medical equipment and supplies as well as support for renovations or redesigns in L&D wards and NCUs.

### 2.3. Variables

The readiness to provide services to small and sick newborns was assessed based on the availability of infrastructure with service delivery rooms in the L&D wards and NCUs, electric power, functional water hygiene and sanitation (WASH) infrastructure, basic amenities, equipment, medications, staffing, and guideline availability.

The variables assessed were the L&D wards and NCUs’ availability of items required for each domain of neonatal care. These included a total of 33 variables across three main domains related to neonatal care in the L&D wards, including 8 variables for basic amenities, 14 for neonatal resuscitation, and 11 for essential newborn care. In the NCUs, 108 variables for 8 domains were assessed, including 16 for basic amenities, 26 for basic equipment, 16 for essential medications, 14 for laboratory investigations, 11 for KMC, 11 for IPC, 6 for staffing—including trained nurses and general practitioners, pediatricians, and support staff (porters, cleaners, and security guards), and 8 for guideline availability.

### 2.4. Data Collection

Prior to the implementation of SLL program interventions, data on hospitals’ readiness to provide care for small and sick newborns were collected by six to eight trained program research assistants in each region. The data collection process was supervised by SLL program evaluation coordinators and regional program managers.

Data collectors and supervisors received two-day training on the data collection tool and methods. Data were gathered using an adapted version 2.2 WHO’s Service Availability and Readiness Assessment (SARA) tool [12]. HCPs and unit heads were interviewed, registers reviewed, and direct observations were conducted of infrastructure in the L&D wards and the NCUs, medical equipment, medications, handwashing facilities, and guideline availability. The data were collected electronically via the Open Data Kit application, transferred to Excel, and thoroughly checked for completeness and accuracy, with corrections being made as needed.

### 2.5. Data Analysis

Data analysis was performed using STATA statistical software, version 17. All categorical variables were summarized using proportions and are presented in tables and graphs. For each item, an item score of 0 or 1 was assigned according to whether the item was available or not at a facility. Next, the percentage of facilities where the item was available was calculated. Readiness scores for each domain were computed as the mean percentage of the items within that domain. A composite readiness score was then calculated by aggregating the domain scores.

The overall readiness was calculated as the mean of the scores for each domain, thus giving equal weight to each of the three domains for the L&D wards and the eight domains for the NCUs. A comparison of item scores within domains across different levels of explanatory variables was conducted. Chi-square tests were used to test for the associations between categorical variables. A *p*-value of <0.05 was considered to indicate a statistically significant association.

## 3. Results

In total 208 public hospitals across all levels were included, 22 (10.5%) referral hospitals, 56 (27.0%) general hospitals, and 130 (62.5%) primary hospitals.

### 3.1. Readiness to Provide Small and Sick Newborn Care in the L&D Wards

**Figure 1:**
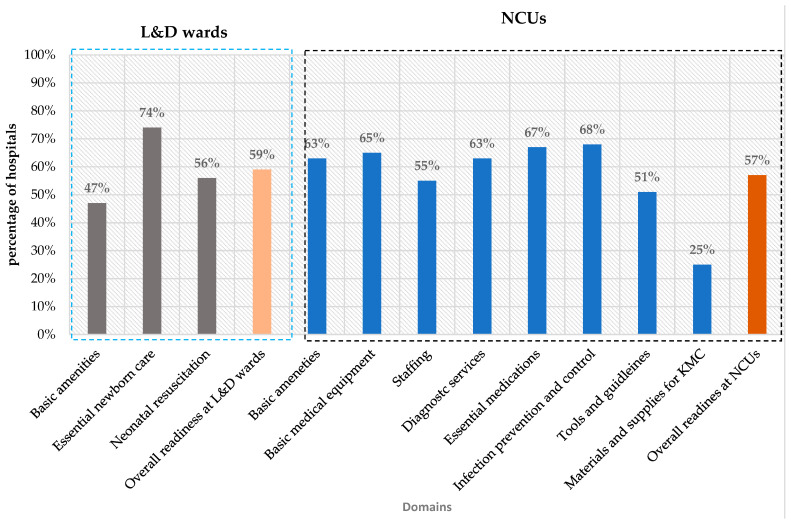
Mean readiness scores by key domains in L&D wards and NCUs.

**Figure 2:**
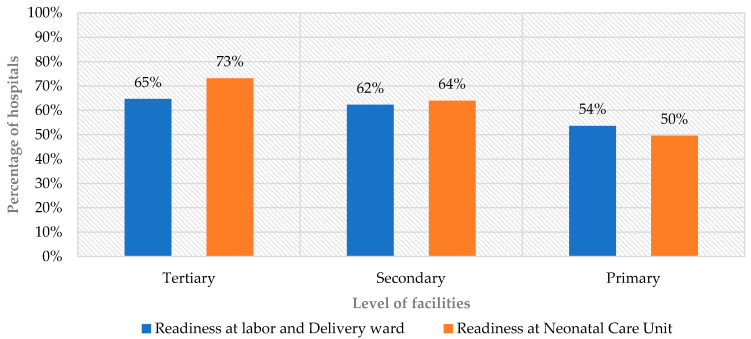
Readiness scores in L&D wards and NCUs by hospital level.

Readiness to provide small and sick newborn care in L&D wards was 47% for basic amenities, 74% for essential newborn care, and 56% for neonatal resuscitation, with an overall mean readiness score of 59% (Figure 1). The overall mean readiness score varied by hospital levels, and referral hospitals scored 65%, general hospitals 62%, and primary hospitals 54% (Figure 2).

The readiness of L&D wards for basic amenities varied by hospital level, with scores of 68% in referral hospitals, 49% in general hospitals, and 43% in primary hospitals. For essential newborn care, the readiness scores were 68% in referral hospitals, 81% in general hospitals, and 71% in primary hospitals (Table 1). Approximately 70% of referral and general hospitals were equipped for neonatal resuscitation, compared to only 50% of primary hospitals (Table 2).

### 3.2. Readiness to Provide Small and Sick Newborn Care in NCUs

The readiness for small and sick newborn care in NCUs was measured across eight domains, including amenities (63%), basic equipment (65%), essential medications (67%), laboratory investigations (63%), KMC (25%), IPC (68%), staffing (55%), and guideline availability (51%). The overall readiness score was 57% (Figure 1) and varied by hospital level: referral hospitals scored 73%, general hospitals 64%, and primary hospitals 50% (Figure 2).

### 3.3. Availability of Basic Amenities in the L&D Wards and NCUs

Functional handwashing facilities were available in 64 (32%) of the prenatal rooms, 105 (50%) of the delivery rooms, 46 (23%) of the postnatal rooms, and 113 (55%) of the NCUs of the assessed hospitals (*p* < 0.05). Functional toilets with showers near the units were available in 106 (51%) of the L&D wards and in 83 (40%) of the NCUs.

Uninterrupted power supply was available in 124 (59%) of L&D wards and 100 (48%) of the NCUs. In the event of a power outage from the national grid, a backup power source was present in 127 (61%) of the hospitals (*p* < 0.05). Uninterrupted water supply was available in only 74 (36%) of the hospitals.

There was a variation in the mean readiness scores for basic amenities across hospital levels in L&D wards and NCUs. In L&D wards, referral hospitals had a mean basic amenities readiness score of 67%, general hospitals 48%, and primary hospitals 46%. In NCUs, referral hospitals had a readiness score of 77%, general hospitals 65%, and primary hospitals 57% (Supplementary Tables S1 and S2).

### 3.4. Availability of Supplies and Equipment for Essential Newborn Care

The readiness scores for essential newborn care also varied by hospital level, with referral hospitals scoring 68%, general hospitals 81%, and primary hospitals 71%. The overall mean readiness score of all the hospital levels was 74% (Table 1).

### 3.5. Availability of Equipment and Supplies for Newborn Resuscitation at L&D Ward

Approximately 90% of hospitals had designated places for neonatal resuscitation. However, only about 56% of hospitals fulfilled basic neonatal resuscitation equipment and supplies. Specifically, only 86 (47%) of the hospitals had neonatal-sized bags and masks, 43 (35%) had self-inflating bags and masks, 68 (54%) had mucus extractors, 66 (47%) had functional oxygen tubing, and 66 (47%) had functional pulse oximeters (*p* < 0.05).

Additionally, standard and/or home-grown bCPAP was available in only 16 (14%) of the hospitals within their L&D wards (*p* < 0.01). The mean readiness score for neonatal resuscitation differed by hospital level and was 68% in referral hospitals, 66% in general hospitals, and 50% in primary hospitals (Table 2).

### 3.6. Readiness for Newborn Care in NCUs

Infrastructure: Nearly all 205 (99%) of the hospitals had designated spaces for NCUs in the hospitals. However, only 69 (59%) of the hospitals had separate rooms dedicated to critically ill, sub-critically ill, and stable infants. The availability of separate rooms in the NCUs varied by hospital level, and 86% of referral hospitals had dedicated spaces for critically ill, sub-critically ill, and stable infants compared to 60% of general hospitals and 47% of primary hospitals (*p* < 0.05).

In addition, most hospitals lacked dedicated rooms within the NCU for triage procedures and did not have an adequate number of beds for mothers (*p* < 0.05) (Supplementary Table S2).

### 3.7. Availability of Medical Equipment and Supplies in NCUs

The overall availability of basic equipment in NCUs was 65%, with wide variation across facility levels: 81% in referral hospitals, 74% in general hospitals, and 59% in primary hospitals. Only 72 (35%) of NCUs had functional bCPAP devices, with availability being the highest in referral hospitals 17 (77%), followed by general hospitals 27 (49%), and primary hospitals 28 (22%) (*p* < 0.05). Less than half of the hospitals 89 (43%) had room thermometers, with approximately 60% of referral and general hospitals compared to only 44 (34%) of primary hospitals.

Additionally, the availability of other essential equipment varied, and room warmers were present in 144 (73%) of hospitals’ NCUs, phototherapy machines in 148 (72%), digital weighing scales in 139 (67%), electrical suction pumps in 107 (53%), pulse oximeters in 156 (75%), and bag-mask self-inflating resuscitators in 122 (60%), *p* < 0.05 (Supplementary Table S3).

### 3.8. Staffing at NCUs

The majority, 188 (90%) of NCUs had nurses trained in neonatal intensive care. Pediatricians were available in 54 (40%) of hospitals, while trained general practitioners were present in 107 (54%) of hospitals. The availability of pediatricians varied significantly by facility level, 95% of referral hospitals, 55% of general hospitals, and only 6% of primary hospitals. The mean readiness score for the availability of key NCU staffing varied by facility level and was 79% in referral hospitals, 56% in general hospitals, and 47% in primary hospitals, with an overall mean of 55% (Supplementary Table S4).

### 3.9. Availability of Essential Medications

A total of 16 essential medications across seven categories were assessed, including antimicrobials, neurologic, inotropes, bronchodilators, corticosteroids, vitamin K, and intravenous infusions for treating small and sick newborns. No health facility reported a complete stock of all 16 medications. The overall readiness score for essential medications was 67%.

More than 90% of hospitals had essential antibiotics such as ampicillin, gentamycin, and ceftriaxone and intravenous fluids and antiseptics, but fewer than half stocked ceftazidime, ciprofloxacin, intravenous azithromycin, or vancomycin.

First-line antimicrobials like ampicillin, gentamicin, and cloxacillin were available in 80% of referral hospitals, 82% of general hospitals, and 81% of primary hospitals. In contrast, second-line drugs were available in only 47% of referral hospitals, 54% of general hospitals, and 33% of primary hospitals. The readiness for essential medications varied across hospital levels at 74% for referral hospitals, 74% for general hospitals, and 61% for primary hospitals (Supplementary Table S5).

### 3.10. Availability of Basic Diagnostic Services

More than two-thirds of the hospitals surveyed could perform essential laboratory tests, including random blood sugar, blood grouping and Rhesus factor, urine analysis, stool examination, and ultrasound. In contrast, fewer than a quarter could perform culture and sensitivity testing, and only 35% offered bilirubin testing or blood morphology assessments.

The mean readiness for laboratory investigations was lower in primary hospitals compared to general and referral hospitals. The overall mean readiness score for laboratory investigations across all hospitals studied was 63% (Supplementary Table S6).

### 3.11. Infection Prevention and Control Supplies

The overall mean readiness score for IPC in the NCUs was 68%. The availability of IPC supplies in NCUs varied across hospitals, and IPC readiness was lower in primary hospitals than in general and referral hospitals. Although alcohol-based hand sanitizers were available in 117 (94%) of the hospitals, the availability of functional handwashing stations differed significantly by facility level at 77% in referral hospitals, 74% in general hospitals, and only 43% in primary hospitals. This disparity highlights the inequitable distribution of basic IPC infrastructure, particularly in lower-level hospitals, which may compromise newborn care and increase the risk of healthcare-associated infections.

Additionally, 91 (74%) of the hospitals had adequate antiseptic solutions, including 7% chlorhexidine, ethanol, povidone-iodine, and disinfectants. Sterile gloves and puncture-proof sharps containers were available in more than three-quarters of the hospitals studied (Supplementary Table S7).

### 3.12. Availability of Guidelines in the NCU

The presence of guidelines varied by guideline type and facility level. Neonatal logbooks were mostly available in 187 (94%) hospitals followed by NCU training guidelines. The IPC national manual, KMC registers, the KMC flow chart/patient chart, feeding and weight charts, preterm care registration books, and preterm care counselling chart booklets were available in fewer than half of the hospitals. The mean readiness for guidelines and tools was 51% at the NCUs of the studied hospitals (Supplementary Table S8).

### 3.13. Availability of Materials and Supplies for KMC Provision

In this study, nearly three-fourths (72%) of the hospitals had designated rooms for KMC. However, among hospitals with KMC rooms, only 31 (16%) had KMC wraps and only 17 (9%) had gowns for mothers (*p* < 0.05). Comfortable chairs were available in 29 (14%) of the hospitals, and reclining beds with privacy curtains were available in 106 (51%) of the hospitals. Additionally, only 16 (8%) of hospitals had televisions to educate mothers on KMC.

The overall readiness for KMC was found to be very low across all facility levels, with a mean readiness score of 25%, with referral hospitals scoring 43%, general hospitals 26%, and primary hospitals 15% (Table 3).

## 4. Discussion

In Ethiopia, neonatal mortality remains high, indicating the urgent need for access to good quality care for all and specialized care for small and sick newborns. This study was conducted to assess the readiness of 208 public hospitals to provide care across the continuum from L&D wards to NCUs. There were significant deficits in readiness, with an overall composite readiness score of 59% in L&D wards and 57% in NCUs. Functional CPAP machines were available in only 14% of L&D wards and in 35% of NCUs. Significant variations in neonatal care readiness were observed across hospital levels, with the lowest readiness in primary hospitals.

### 4.1. Basic Amenities in L&D Wards and NCUs

Forty seven percent of the hospitals had key basic amenities for newborn care in the L&D wards, and this varied by hospital level, ranging from 68% in referral hospitals to 43% in primary hospitals. Safe water, sanitation and hygiene (collectively known as WASH) is essential for proper IPC practices in preventing neonatal infections [13]. Lack of water supply hamper hand hygiene, instrument cleaning, service delivery, and overall clinical safety. The current study showed that most hospitals, across all levels of care, lacked continuous water supplies in L&D wards and NCUs. This finding is consistent with the Ethiopian service provision assessment 2021–2022, which reported that only half of facilities had a continuous water supply [14].

In the current study, only half of the hospitals had uninterrupted electricity, with frequent and prolonged outages, particularly in primary hospitals. Such power disruptions can negatively affect sick and small newborns health care service, leading to severe complications or death. Comparable findings have previously been reported from Ethiopia and other low and middle income countries, where power interruptions remain a persistent barrier to safe neonatal care [14,15]. In contrast, studies from India, showed more consistent electric power supply in higher-level hospitals, although rural and peripheral facilities continued to face challenges [16,17].

### 4.2. Readiness for Essential Newborn Care and Respiratory Support

Regarding hospitals’ readiness for essential newborn care, about three-quarters of hospitals were well prepared. This was higher than that reported from public health facilities in Afghanistan where fewer than half of had essential supplies [18]. Hospitals are expected to have a dedicated space for neonatal resuscitation and to be fully equipped with essential devices and supplies. Although nearly 90% of the hospitals in our study had designated resuscitation corners, none were fully equipped with the complete set of recommended devices and materials. This finding aligns with other studies from low- and middle-income countries and a study done on the quality of neonatal resuscitation in Ethiopia reporting that only half of health facilities were adequately prepared for neonatal resuscitation in terms of essential equipment [19,20]. Similarly, a facility audit in southern Nigeria reported significant gaps in basic resuscitation devices [21]. These gaps emphasize the need to equip health facilities with the basic devices needed to support newborns with respiratory problems to improve neonatal outcomes.

Almost 10% of newborns and a large proportion of preterm infants cannot breathe spontaneously at birth and require bag-mask ventilation [22]. However, we found that basic equipment for bag-mask ventilation was lacking in half of the hospitals studied. This finding contrasts with a previous study from Ethiopia, which reported that more than 70% of hospitals had neonatal bags and masks [23].

The WHO recommends the use of bCPAP for preterm newborns with signs of RDS and for all infants born before 32 weeks of gestation [24]. Nearly 11% of newborns in Ethiopia are born preterm, and may require respiratory support with bCPAP [25]. However, we found that only 14% of L&D wards and 35% of NCUs had either standard or improvised bCPAP machines. Low availability of bCPAP has also been reported studies from other African countries [26].

### 4.3. Readiness Regarding Amenities, Equipment, and Drugs in NCUs

Effective neonatal care in NCUs requires adequate equipment and supplies as well as skilled HCPs [27]. Our findings showed significant gaps across all domains, with an overall readiness score of about 60%. Although two-thirds of hospitals had basic amenities, medicines, equipment, lab tests, and IPC supplies, only half had the required staffing and guidelines. Similar gaps in neonatal readiness have been reported from India, especially in lower-level hospitals [28].

Regarding the availability of equipment in NCUs, no facility had all the required basic equipment, and overall, nearly two thirds of hospitals were well equipped with NCU beds, radiant warmers, room warmers, oxygen cylinders, portable electrical suction pumps, oxygen concentrators, digital thermometers, and pulse oximeters. Compared to another study from Ethiopia, where nearly one quarter of the hospitals met the standards, there has been some progress at higher levels, though overall preparedness remains suboptimal [29]. In the current study, nearly two-thirds of hospitals had phototherapy machines and 92% had functional radiant warmers. This was similar to findings from Tanzania and Pakistan [27,30].

The availability of essential drugs is critical for managing infections and for saving sick newborns. However, our study identified significant gaps across hospitals, with none reporting the availability of all 16 assessed drugs, including antimicrobials, neurologic agents, inotropes, bronchodilators, corticosteroids, vitamin K, and IV infusions at the time of the study. Approximately four-fifths of hospitals had first-line antimicrobials, including ampicillin, gentamicin, and cloxacillin. However, second-line drugs such as ceftriaxone, ceftazidime, ciprofloxacin, IV azithromycin, and vancomycin were available in only 33–54% of hospitals. Similar shortages have been reported in the findings from Pakistan, indicating widespread challenges in ensuring access to essential medications for newborns [30].

Regarding the readiness of hospitals to perform laboratory investigations, our findings showed that two thirds of the hospitals had basic laboratory services. Readiness levels varied by facility type, ranging from 74% in referral hospitals to 58% in primary hospitals. The readiness of primary hospitals in our study was comparable to findings from similar study done on the readiness of primary hospitals in providing neonatal intensive care services in Ethiopia, which reported 65% readiness for different laboratory services [29].

### 4.4. KMC

Despite the WHO’s strong recommendation of KMC for all preterm and LBW infants [24], most hospitals in our study showed low readiness. Although three-quarters of facilities had dedicated KMC rooms, most were not equipped with essential materials and supplies for KMC. Similar gaps have been reported in other Ethiopian studies [31].

### 4.5. Human Resources

Trained nurses were available in nearly all studied hospitals, indicating relatively higher readiness compared to another Ethiopian study, where only 65% of hospitals had trained neonatal HCPs [32]. Despite overall staffing shortages, this suggests the increased availability of trained nurses in NCUs.

Pediatricians play a key role in neonatal care and help train and guide other staff. However, in our study fewer than half of the hospitals had pediatricians on their staffing, with most being based in referral hospitals, which contrasts with findings from Pakistan, where all hospitals reported having pediatricians [30] and aligns with other studies in south Nigeria and Ethiopia [21,33].

### 4.6. Infection Prevention and Control

Evidence indicates that effective IPC measures can reduce healthcare-associated infections by up to 55%, and newborn survival rates can potentially increase by 44% when hand washing and clean birthing kits are in place [13]. The WHO recommends that all health facilities should follow minimum IPC standards at the point of care, including continuous water supply, hand hygiene supplies, disinfectants, proper waste management, and adequate space for IPC practices [34]. However, only about 70% of the hospitals in this study had the necessary IPC supplies. About half of the hospitals had functional handwashing facilities and 75% had essential antiseptics and disinfectants. These findings align with a study done in seven countries in East Asia and the Pacific concluding that coverage of IPC services must be improved to reduce the risk of neonatal mortality and morbidity [35].

### 4.7. Strengths and Limitations of the Study

Strengths of this study was the population-based design including 208 hospitals from four large regions of Ethiopia, and that a standardized tool was used for data collection.

However, among the 290 hospitals identified for participation in the SLL program, 82 hospitals were excluded due to security reasons, remote location or financial constraints. This may have overestimated the overall readiness to provide neonatal services in Ethiopia.

Lastly, we recognize that neonatal outcomes are dependent on maternal follow-up in pregnancy and during labour. However, assessment of maternal care was outside the scope of this study.

## 5. Conclusions

Despite improvements in recent years, we found many gaps in critical readiness domains in L&D wards and NCUs. The government and partners along with other stakeholders must collaborate to ensure the consistent availability of sufficient amenities, equipment, essential diagnostic and treatment commodities, staffing, and guidelines for high-quality newborn care at all hospital levels.

Moreover, we highly recommend regular readiness assessments of hospitals for neonatal care to ensure early gap identification and evidence-based solutions to enhance the quality of newborn care and thus improve survival outcome for preterm and LBW infants.

## Figures and Tables

**Table 1:** Availability of basic supplies and equipment for essential newborn care at the L&D ward by hospital level.

Variables	Tertiary		General		Primary		Overall		*p*-Value
	*n*	*n* (%)	*N*	*n* (%)	*N*	*n* (%)	*N*	*n* (%)	
Vitamin K	22	19 (86)	56	51 (91)	130	108 (83)	208	178 (86)	0.36
TTC eye ointment	22	19 (86)	56	51 (91)	130	116 (89)	208	186 (89)	0.82
Chlorhexidine 4% gel	21	14 (67)	51	34 (67)	126	85 (67)	198	133 (67)	0.99
Baby weighing scale	17	16 (94)	51	49 (96)	123	119 (97)	191	184 (96)	0.85
Sterile scissors and/or blades	17	16 (94)	27	27(100)	81	81 (100)	125	124 (99)	0.04
Umbilical cord clamp	17	15 (88)	27	26 (96)	81	77 (95)	125	118 (94)	0.48
Clean blankets, towels, and linens	17	3 (18)	27	15 (56)	81	38 (47)	125	56 (45)	0.03
Wall clock	22	9 (41)	55	37 (67)	127	46 (36)	204	92 (45)	0.00
Measuring tape	18	10 (56)	52	39 (75)	119	60 (50)	189	109 (58)	0.01
Stethoscope	20	17 (85)	50	42 (84)	100	63 (63)	170	122 (72)	0.01
Baby crib	7	2 (29)	39	28 (72)	91	49 (54)	137	79 (58)	0.04
Mean readiness score		68		81		71		74	

**Table 2:** Neonatal resuscitation space, equipment, and supply availability overall and by hospital level in L&D wards.

Variables	Referral		General		Primary		Overall		*p*-Value
	*n*	*n* (%)	*N*	*n* (%)	*N*	*n* (%)	*N*	*n* (%)	
Space/corner	22	20 (91)	56	51 (91)	130	115 (88)	208	186 (89)	0.84
Warmer	18	15 (83)	38	29 (76)	83	72 (87)	139	116 (83)	0.36
Suction pump (electrical)	21	10 (48)	42	26 (62)	93	38 (41)	156	74 (47)	0.08
Suction pump (manual)	22	20 (91)	55	44 (80)	129	96 (74)	206	160 (78)	0.20
Functional oxygen cylinder	16	16 (100)	27	20 (74)	81	51(63)	124	87 (70)	0.01
Functional oxygen concentrator	16	9 (56)	52	45 (87)	118	83 (70)	186	137 (74)	0.02
bCPAP machine and/or indigenous	16	5 (31)	27	10 (37)	75	1 (1)	118	16 (14)	0.00
Neonatal size bag, self-inflating	22	13 (59)	56	38 (68)	104	35 (34)	182	86 (47)	0.00
Neonatal size face masks (size 0–1)	16	14 (88)	28	25 (89)	56	43 (77)	100	82 (82)	0.31
Nasal prongs, 1 mm and 2 mm	17	10 (59)	27	16 (59)	87	37 (43)	131	63 (48)	0.20
Laryngoscope, neonatal size	12	4 (33)	11	2 (18)	59	7 (12)	82	13 (16)	0.17
Mucus extractor	16	11 (69)	27	16 (59)	79	16 (20)	122	43 (35)	0.00
Oxygen tubing	17	11 (65)	28	20 (71)	81	37 (46)	126	68 (54)	0.04
Functional pulse oximeter	17	13 (76)	28	14 (50)	96	39 (41)	141	66 (47)	0.02
Mean readiness score		68		66		50		56	

**Table 3:** Availability of materials and supplies for KMC service by hospital level.

Items	Referral		General		Primary		Overall		
	*n*	*n* (%)	*N*	*n* (%)	*N*	*n* (%)	*N*	*n* (%)	*p*-Value
Dedicated space for KMC	21	20 (95)	51	35 (67)	126	88 (70)	198	143 (72)	0.04
KMC wraps	21	5 (24)	51	9 (18)	126	17 (13)	198	31 (16)	0.44
Gowns for mothers	21	5 (24)	51	2 (4)	126	10 (8)	198	17 (9)	0.02
TV for health education	21	7 (33)	51	2 (2)	126	7 (6)	198	16 (8)	0.00
Reclining beds with curtains	22	16 (73)	56	28 (50)	130	62 (48)	208	106 (51)	0.09
Comfortable chairs	22	7 (31)	56	6 (11)	129	16 (12)	207	29 (14)	0.04
Cabinets for mothers/bed side	22	9 (40)	56	15 (27)	130	19 (15)	208	43 (21)	0.01
Refrigerator	21	9 (43)	51	7 (14)	126	8 (6)	198	24 (12)	0.00
Room warmer	21	8 (38)	51	23 (45)	111	32 (29)	198	63 (34)	0.12
Food for mothers	21	16 (76)	45	21 (47)	111	25 (23)	177	62 (35)	0.00
Mean score		43		26		15		25	

## Data Availability

Data are available on reasonable request.

## Supplementary Materials

The following supporting information can be downloaded at: https://www.mdpi.com/article/10.3390/children13040481/s1, Table S1: Availability of basic amenities in the labour & delivery ward by hospital level; Table S2: Availability of basic amenities in NCUs by hospital level; Table S3: Availability of basic medical equipment at NCUs by hospital level; Table S4: Key staffing availability at the NCU by hospital level; Table S5: Availability of basic medications in the NCU by hospital level; Table S6: Availability of basic diagnostic services by hospital level; Table S7: Availability of infection prevention supplies at the NCU by hospital level; Table S8: Availability of guidelines at the NCU by hospital level.

## Abbreviations

bCPAP	Bubble continuous positive airway pressure
HCPs	Health care providers
IPC	Infection prevention and control
KMC	Kangaroo Mother Care
LBW	Low birth weight
L&D	Labour and delivery
NCUs	Neonatal care units
SARA	Service Availability Readiness Assessment
SLL	Saving Little Lives
WASH	Water, sanitation, and hygiene
