# Supplementary material for: Readiness to Provide Neonatal Care Services in 208 Ethiopian Hospitals Prior to Implementation of the Saving Little Lives Program
Source: Children (Basel). 2026 Mar 30;13(4):481. doi: 10.3390/children13040481 (PMC13115033; doi:10.3390/children13040481)
Supplement: Supplementary file 1 [file children-13-00481-s001.zip › children-4130955-supplementary.pdf]

Online Supplementary Documents

Health Facilities' Service Availability and Readiness for Neonatal Care in Ethiopian Hospitals - the Saving Little Lives program

Table S1. Availability of basic amenities in the labour & delivery ward by hospital level .

	**Referral**		**General**		Primary		Overall		p-value
**Variables**	**n**	**n (%)**	**n**	n (%)	n	n (%)	n	n (%)	
Functional hand washing stations in:									
Prenatal room	21	14 (67)	51	16 (31)	126	34 (27)	198	64 (32)	0.00
Delivery room	22	17 (77)	56	28 (50)	130	60 (46)	208	105 (50)	0.03
Postnatal room	21	10 (48)	51	9 (18)	126	27 (21)	198	46 (23)	0.02
Curtains & bed screens in each bed in:									
Prenatal room	21	9 (43)	51	14 (27)	82	21(26)	154	44 (29)	0.29
Delivery room	22	12 (55)	56	25 (45)	130	54 (42)	208	91 (44)	0.52
Postnatal room	21	13 (62)	51	12(24)	82	10 (12)	154	35 (23)	0.00
Nursing station	21	20 (95)	51	41 (80)	126	109 (87)	198	170 (86)	0.24
Toilet with shower adjacent to ward	22	12 (55)	56	31(55)	130	63 (48)	208	106 (51)	0.65
Uninterrupted electricity supply	22	17 (77)	56	38 (68)	130	45 (35)	208	100 (48)	0.00
Automatic backup generator	22	20 (91)	56	34 (61)	130	73 (56)	208	127 (61)	0.01
Uninterrupted water supply	22	13 (59)	56	31 (55)	130	30 (23)	208	74 (36)	0.00
Single entrance to control access	21	17 (81)	51	37 (73)	124	112 (90)	196	166 (85)	0.01
**Mean readiness score**		**68**		**49**		**43**		**47**	

Table S2. Availability of basic amenities in NCUs by hospital level.

	**Tertiary**		General		Primary		Overall		
**Variables**	**n**	**n (%)**	n	n (%)	n	n (%)	n	n (%)	p-value
Separate space for NCU	22	22 (100)	56	56 (100)	130	127 (98)	208	205 (99)	0.40
NCU is located adjacent to delivery room	21	11 (52)	51	29 (57)	123	110 (89)	195	150 (77)	0.00
Direct access to transport receiving area	17	13 (76)	34	19 (56)	17	14 (82)	68	46 (68)	0.11
Rooms for critical, sub-critical, and stable cases	21	18 (86)	45	27 (60)	51	24 (47)	117	69 (59)	0.01
Triaging room	21	18 (86)	51	22 (43)	42	5 (12)	114	45 (39)	0.00
Procedure room	13	10 (76)	43	22 (51)	16	5 (31)	72	37 (51)	0.05
IV drugs and fluid preparation area	22	18 (82)	56	42 (75)	130	92 (71)	208	152 (73)	0.52
Gowning area at the entrance	21	15 (71)	48	32 (67)	94	60 (64)	163	107 (66)	0.79
Nurses station/staff work area	22	15 (68)	54	39 (72)	122	94 (77)	198	148 (75)	0.59
Workspace for doctor/nurse on duty	22	18 (82)	56	37 (66)	65	36 (55)	143	91 (64)	0.07
One or two rooms with 4–6 maternal beds	21	16 (76)	51	33 (65)	79	23 (29)	151	72 (48)	0.00
Uninterrupted water supply in each room	22	13 (59)	56	31 (55)	130	30 (23)	208	74 (36)	0.00
Functional hand washing facilities	22	17 (77)	54	40 (74)	130	56 (43)	206	113 (55)	0.00
Toilet and shower area	22	15 (68)	55	18 (33)	130	50 (38)	207	83 (40)	0.01
Uninterrupted 24 h stabilized power supply	22	17 (77)	56	41 (73)		66 (51)	208	124 (60)	0.00
24 h service availability	22	22 (100)	54	50 (93)	130	127 (98)	206	199 (97)	0.14
**Mean readiness score**		**77**		**65**		**57**		**63**	

Table S3. Availability of basic medical equipment at NCUs by hospital level.

	**Referral**		**General**		Primary		Overall		
**Items**	**n**	**n (%)**	**n**	n (%)	n	n (%)		n (%)	p-value
NCU Bed	22	22 (100)	45	43 (96)	129	122 (95)	196	187 (95)	0.53
Radiant warmers	22	22 (100)	56	54 (96)	130	116 (89)	208	192 (92)	0.09
Room warmers	21	11 (52)	4-	40 (80)	126	93 (74)	197	144 (73)	0.05
Room thermometer	22	13 (59)	55	32 (58)	130	44 (34)	207	89 (43)	0.00
Oxygen cylinder, 20 litres	21	20 (95)	40	35 (88)	120	112 (93)	181	167 (92)	0.42
Phototherapy	22	21 (95)	55	48 (87)	128	79 (62)	205	148 (72)	0.00

Neonatal resuscitator, hand operated, 250 ml	11	8 (73)	32	23 (72)	77	33 (43)	120	64 (53)	0.01
Suction bulb	22	17 (77)	56	43 (77)	128	88 (69)	206	148 (72)	0.45
Portable electrical suction pump	18	16 (89)	53	35 (66)	130	56 (43)	201	107 (53)	0.00
IV infusion pump	22	19 (86)	56	32 (57)	130	36 (28)	208	87 (42)	0.00
Syringe pump	22	20 (91)	50	38 (76)	124	84 (68)	196	142 (72)	0.07
Oxygen concentrators	21	18 (86)	49	43 (88)	130	111 (85)	200	172 (86)	0.92
Incubators	22	19 (86)	56	47 (84)	129	97 (75)	207	163 (79)	0.27
Glucometer	22	21 (95)	55	44 (80)	130	93 (72)	207	158 (76)	0.04
Digital thermometer, clinical	22	20 (91)	54	50 (93)	129	121 (93)	205	191 (93)	0.87
Weighing scale neonate (Digital)	22	17 (77)	54	42 (78)	130	80 (61)	206	139 (67)	0.06
Pulse oximeter, neonatal	22	20 (91)	56	48 (85)	129	88 (68)	207	156 (75)	0.01
Stethoscope, neonatal	15	12 (80)	45	36 (80)	130	69 (53)	190	117 (62)	0.00
Tape measure, vinyl-coated, 1.5 m	15	8 (53)	45	32 (71)	124	80 (64)	184	120 (65)	0.44
Infusion stands	14	13 (93)	50	41 (82)	112	85 (75)	176	139 (79)	0.28
bCPAP with a compressor	22	17 (77)	55	27 (49)	130	28 (22)	207	72 (35)	0.00
Gown and shoes for the providers	21	15 (71)	51	29 (57)	120	59 (49)	192	103 (54)	0.15
Refrigerator for drugs	22	20 (90)	55	28 (51)	130	12 (9)	207	60 (29)	0.00
Bag and mask, self-inflating	22	14 (63)	53	42 (79)	130	66 (51)	205	122 (60)	0.00
Laryngoscope	19	13 (68)	45	22 (49)	105	17 (16)	169	52 (31)	0.00
Mobile examination lights, ≥ 2	19	11 (58)	48	23 (48)	120	47 (39)	187	81 (43)	0.24
**Mean readiness score**		**81**		**74**		**59**		**65**	

Table S4. Key staffing availability at the NCU by hospital level

	Referral		General		Primary		Overall		
**Items**	n	n (%)	n	n (%)	n	n (%)	n	n (%)	p-value
NCU trained nurses	22	21 (95)	56	54 (96)	130	113 (87)	208	188 (90)	0.09
Paediatricians	22	21 (95)	55	30 (55)	57	3 (6)	134	54 (40)	0.00
Trained general practitioner	19	11(58)	56	31 (55)	124	65 (52)	199	107 (54)	0.87
Porters	22	18 (82)	43	11 (25)	124	42 (33)	189	71 (38)	0.00
Cleaners	22	21 (95)	39	34 (87)	120	100 (83)	181	155 (86)	0.31
Guards	22	11(50)	54	9 (16)	124	28 (23)	200	48 (24)	0.01
**Mean readiness score**		**79**		**56**		**47**		**55**	

Table S5. Availability of basic medications in the NCU by hospital level

	**Referral**		**General**		Primary		Overall		
**Drugs**	**n**	**n (%)**	**n**	n (%)	n	n (%)	n	n (%)	p-value
Ampicillin	21	20 (95)	50	46 (92)	126	123 (98)	197	189 (96)	0.23
Gentamycin	22	18 (82)	51	49 (96)	126	115 (91)	199	182 (91)	0.13
Ceftriaxone	21	17 (81)	50	46 (92)	126	114 (90)	197	177 (90)	0.35
Cloxacillin	21	13 (62)	40	23 (58)	126	68 (54)	187	104 (56)	0.77
Ceftazidime	14	4 (29)	40	17 (43)	120	41 (34)	174	62 (36)	0.54
Ciprofloxacin	14	7 (50)	41	26 (63)	50	5 (10)	105	38 (36)	0.00
Azithromycin IV	14	2 (14)	34	5 (15)	50	2 (4)	98	9 (9)	0.19
Vancomycin	13	8 (62)	34	20 (59)	50	14 (28)	97	42 (43)	0.01
Tetracycline eye ointment	20	13 (77)	51	44 (86)	126	95 (75)	197	152 (77)	0.12
Vitamin K ampoules	21	16 (76)	51	45 (88)	126	86 (68)	198	147 (74)	0.02
IV fluids (N/S, 5% D/W, 40% glucose)	21	21 (100)	51	48 (94)	124	116 (94)	196	185 (94)	0.49
Antiseptics	21	19 (90)	51	49 (96)	125	120 (96)	197	188 (95)	0.52
Adrenaline amp.	13	12 (92)	32	26 (81)	35	18 (51)	80	56 (70)	0.01
Aminophylline amp.	14	14 (100)	34	25 (74)	50	22 (44)	98	61(62)	0.00
Dexamethasone injection	14	14 (100)	40	33 (83)	85	63 (74)	139	110 (79)	0.07
Phenobarbital	14	11 (79)	40	24 (60)	119	70 (59)	173	105 (61)	0.36
**Mean score**		**74**		**74**		**61**		**67**	

Table S6 Availability of basic diagnostic services by hospital level

Items	Referral		General		Primary		Overall		
	**n**	**n (%)**	**n**	n (%)	n	n (%)		n (%)	p-values
HCT or Hgb	18	14 (78)	40	30 (75)	104	73 (70)	162	117 (72)	0.73
CBC	18	14 (78)	40	28 (70)	105	56 (53)	163	98 (60)	0.05
Random blood sugar	16	16 (89)	40	31 (78)	105	83 (79)	163	130 (80)	0.58
Blood group & Rh factor	11	9 (82)	40	30 (75)	105	80 (76)	156	119 (76)	0.89
Bilirubin total & direct	10	6 (60)	34	19 (56)	52	8 (15)	96	33 (34)	0.00
Blood Morphology	7	4 (57)	34	11 (32)	11	3 (27)	52	18 (35)	0.39
Blood Film	11	10 (90)	40	31 (78)	82	52 (63)	133	93 (70)	0.08
ESR	11	6 (55)	40	28 (70)	82	39 (48)	133	73 (55)	0.06
VDRL	10	7 (70)	40	29 (73)	105	73 (70)	155	109 (70)	0.94
Urine analysis	11	8 (73)	40	36 (90)	105	79 (75)	156	123 (79)	0.13
Stool exam	11	8 (73)	40	38 (95)	105	81 (77)	156	127 (81)	0.04
Culture and sensitivity of any fluid	5	5 (71)	34	5 (15)	11	0	52	10 (19)	0.00
X-Ray	15	11 (73)	34	27 (79)	50	32 (64)	99	70 (71)	0.30
Ultrasound	13	12 (92)	36	20 (56)	34	30 (88)	83	62 (75)	0.00
**Mean readiness score**		**74**		**67**		**58**		**63**	

Table S7. Availability of infection prevention supplies at the NCU by hospital level

Items	Referral	General	Primary	Overall	p-value
	n = 16	n = 27	n = 81	n = 124	
	n (%)	n (%)	n (%)	n (%)	
**Alcohol-based hand rub**	15 (94)	27 (100)	75 (93)	117 (94)	0.35
Adequate antiseptics (chlorhexidine 7%, ethanol, povidone-iodine)	15 (94)	19 (70)	57 (70)	91 (74)	0.14
Functional hand washing facilities	15 (94)	27 (100)	25 (31)	67 (54)	0.00
Contaminated waste bins with different colors	11 (68)	21 (79)	37 (46)	69 (55)	0.01
Disinfectant solutions (e.g. chlorine bleach)	16 (100)	25 (92)	70 (86)	111 (90)	0.23
Adequate disposable gloves	14 (87)	20 (74)	41 (50)	75 (60)	0.01
Gloves (sterile)	15 (94)	24 (89)	52 (64)	91 (73)	0.01
Regular trash/waste bin	13 (81)	26 (96)	36 (44)	75 (60)	0.00
Receptacle for soiled linen	10 (63)	19 (70)	24 (29)	53 (43)	0.00
Sharps containers (puncture proof)	14 (88)	24 (89)	74 (91)	112 (90)	0.86
Autoclave	6 (38)	22 (81)	36 (44)	64 (52)	0.00
**Mean readiness score**	**82**	**86**	**59**	**68**	

Table S8. Availability of guidelines at the NCU by hospital level

Items	Referral		General		Primary		Overall		
	**n**	**n (%)**	**n**	n (%)	n	n (%)	n	n (%)	p-value
NCU training guideline	15	12 (80)	25	23 (92)	75	49 (65)	115	84 (73)	0.03
IPC national manual	11	10 (91)	21	11 (52)	49	17 (34)	81	38 (47)	0.00
KMC registers	15	7 (47)	26	13 (50)	75	8 (11)	116	28 (24)	0.00
KMC flow chart/patient chart; feeding and weight	20	14 (70)	55	37 (67)	130	48 (36)	205	99 (48)	0.00
Neonatal logbook	21	20 (95)	54	51 (94)	123	116 (94)	198	187 (94)	0.99
Preterm care registration book	17	11 (65)	40	17 (43)	79	16 (20)	136	44 (32)	0.00
Preterm care counselling chart booklet	14	8 (57)	42	17 (40)	94	11 (12)	150	36 (24)	0.00
MPDSR audit	12	12 (100)	25	18 (72)	75	44 (59)	112	74 (66)	0.02
**Mean readiness score**		**75.6**		**63.8**		**41.4**		**51.0**	
